# Effects of physiological integration on nitrogen use efficiency of moso bamboo in homogeneous and heterogeneous environments

**DOI:** 10.3389/fpls.2023.1203881

**Published:** 2023-06-13

**Authors:** Jiancheng Zhao, Chunju Cai

**Affiliations:** ^1^ Zhejiang Provincial Key Laboratory of Bamboo Research, Northwest Zhejiang Bamboo Forest Ecosystem Positioning Observation and Research Station, Zhejiang Academy of Forestry, Hangzhou, Zhejiang, China; ^2^ Key Laboratory of National Forestry and Grassland Administration/Beijing Bamboo & Rattan Science and Technology, International Centre for Bamboo and Rattan, Beijing, China

**Keywords:** *Phyllostachys edulis*, physiological integration, clonal plant, N translocation, NUE

## Abstract

**Introduction:**

Moso bamboo is one of the important clonal plants with complex underground rhizome-root system. Ramets connected by rhizome can translocate and share nitrogen (N), which may affect the nitrogen use efficiency (NUE) of moso bamboo. The aims of this study were to investigate the mechanisms of N physiological integration and its relationship with NUE of moso bamboo.

**Methods:**

A pot experiment was conducted to trace the movement of ^15^N between the connected ramets of moso bamboo in both homogeneous and heterogeneous N environments.

**Results:**

Results showed that N translocation within clonal fragments of moso bamboo was detected in both homogeneous and heterogeneous environments. The intensity of physiological integration (IPI) was significantly lower in homogeneous environments than that in heterogeneous environments. ^15^N translocation between the connected ramtes of moso bamboo was determined by the source-sink relationship in heterogeneous environments, and the ^15^N allocation of the fertilized ramet was higher than that of the connected unfertilized ramet. The NUE of connected treatment was significantly higher than that of severed treatment, which suggested that physiological integration significantly improved the NUE of moso bamboo. In addition, the NUE of moso bamboo was significantly higher in heterogeneous environments than that in homogeneous environments. The contribution rate of physiological integration (CPI) on NUE in heterogeneous environments was significantly higher than that in homogenous environments.

**Discussion:**

These results will provide theoretical basis for precision fertilization in moso bamboo forests.

## 1 Introduction

Bamboo, the fast-growing grass plant, is a precious treasure bestowed upon humankind by nature and has significant ecological, economic, and social benefits (Jiang, 2007). There are 1642 bamboo species in the world and the area of bamboo forest is more than 35 million ha (Vorontsova et al., 2017). Bamboo culms are connected with each other through the rhizome-root system, with strong physiological integration functions and environmental adaptability (Zhuang et al., 2011). Physiological integration is an important characteristic of clonal plants, that refers to the translocation and sharing of photosynthates, water and mineral nutrients between the connected ramets by rhizomes, stolons, or roots (He et al., 2010; Portela et al., 2021; Shi et al., 2021; Wang et al., 2021; Li et al., 2022; Shi et al., 2022).

Moso bamboo (*Phyllostachys edulis*) is an important economic bamboo species for the production of bamboo timbers and bamboo shoots, which is widely distributed in southern China (Song et al., 2011; Shi et al., 2022; Zhao et al., 2022). The 9th national forest resources inventory shows that there is 4.68 million ha of moso bamboo forest, accounting for 72.96% of the total area of bamboo forest (National Forestry and Grassland Administration, 2019). As a group of typical clonal plants, moso bamboo has many unique properties that can effectively utilize resources in heterogeneous habitats through physiological integration (Zhuang et al., 2011). The integration between connected ramets can transfer nutrients to each other and then increase the net growth rate of the population.

Moso bamboo forest is also a typical uneven-aged forest composed of individuals of different ages due to its unique growth characteristics and conventional managements (Su et al., 2019; Shi et al., 2022). The ages of bamboo culms are expressed by “du” due to the growth cycle of two years (on-year and off-year) in moso bamboo forests (Su et al., 2019; Zhao et al., 2019; Li et al., 2021). In “on-year”, more than 90% of new shoots are produced, followed by a few new shoots in “off-year” (Tang et al., 2015). In addition, the growth of moso bamboo in both diameter and height is completed within two months after shoot sprouting, which is referred to as “explosive growth” (Song et al., 2016; Shi et al., 2022). During the “explosive growth” period, the nutrients for the growth of new individuals are supplied by the connected older individuals through rhizome (Sun et al., 2019), which has important ecological significance for the survival, growth, reproduction, expansion and resource utilization of moso bamboo (He et al., 2010; Portela et al., 2021; Wang et al., 2021; Li et al., 2022).

The special management measures in moso bamboo forest, such as bamboo timbers cutting and bamboo shoots harvesting, brought away large amount of nutrients, leading to an unsustainable level of long-term productivity (Guan et al., 2017; Su et al., 2019). Numerous studies showed that nitrogen (N) had the largest demand in moso bamboo forests (Su, 2012; Zhao et al., 2016). Therefore, the nitrogenous fertilizers were commonly applied in moso bamboo forests (Su et al., 2019). Mao et al. (2016) investigated the NUE of moso bamboo forests in homogeneous environments by broadcast application, and the N use efficiency (NUE) was relatively low. In order to improve the NUE of moso bamboo forests, furrow application and hole application were used to determine the appropriate fertilization placement and the target age (Su et al., 2019). What’s more, cavity-injecting fertilization was conducted as heterogeneous environments, and N allocation in bamboo individuals of different ages was also conducted (Shi et al., 2022). Their results showed that the N competitive ability and NUE of I “du” (1–2 years old) bamboo were significantly higher than II “du” (3–4 years old) and III “du” (5–6 years old) bamboos (Zhao, 2016; Su et al., 2019). In addition, the unequal N translocation pattern caused by physiological integration between two connected ramets ensured necessary N supply for young moso bamboo growth, and the demand-driven source-sink relationships significantly affected N translocation in the clonal fragment of moso bamboo (Shi et al., 2022). These results mainly focused on N translocation between parent and offspring ramets, while little information was known between two connected ramets with the same age.

In this study, we sought to investigate the characteristics of N physiological integration between two connected ramets with the same age and the effect of physiological integration on the NUE of moso bamboo. To do this, we conducted a pot experiment using the clonal fragments of moso bamboo with the same age in both homogeneous and heterogeneous environments. Fertilizers (urea or ^15^N-labeled urea) were applied, and rhizomes between two successive ramets were either connected or severed. We aimed to answer the following specific questions: (1) Is translocation of N in clonal fragments different between homogeneous and heterogeneous environments? (2) Dose physiological integration improve the NUE of moso bamboo forests in both homogeneous and heterogeneous environments?

## 2 Materials and methods

### 2.1 Materials and experimental design

The experiment was conducted in Bamboo Botanical Garden (120°03′42′′E, 30°22′25′′N), Zhejiang Academy of Forestry, China. In April 2019, the seedlings of moso bamboo were cultivated by semination technology. In May 2021, fifty healthy rhizomes were selected and cut into 0.5 m, which had more than two rhizome buds. Then, the selected rhizomes were placed on the seedbed and covered with a thin layer of cultivation substrate. The cultivation substrate was a mixture of red soil and fine sand by volume (3:1), with a pH value of 5.83, organic carbon (C) concentration of 26.47 g kg^−1^, N concentration of 1.59 g kg^−1^, P concentration of 0.48 g kg^−1^, and K concentration of 14.25 g kg^−1^. In addition, proper water was applied to keep moist, so as to promote the emergence of the rhizome buds as soon as possible. In May 2022, the clonal fragments with two successive ramets connected by rhizomes were selected and transplanted in two non-woven bags (30 cm in diameter, 30 cm in depth). The average height and ground diameter of ramets were 81.58 cm and 4.46 mm, respectively. The clonal fragments were placed under a canopy in order to avoid rainfall interference.

**Figure 1:**
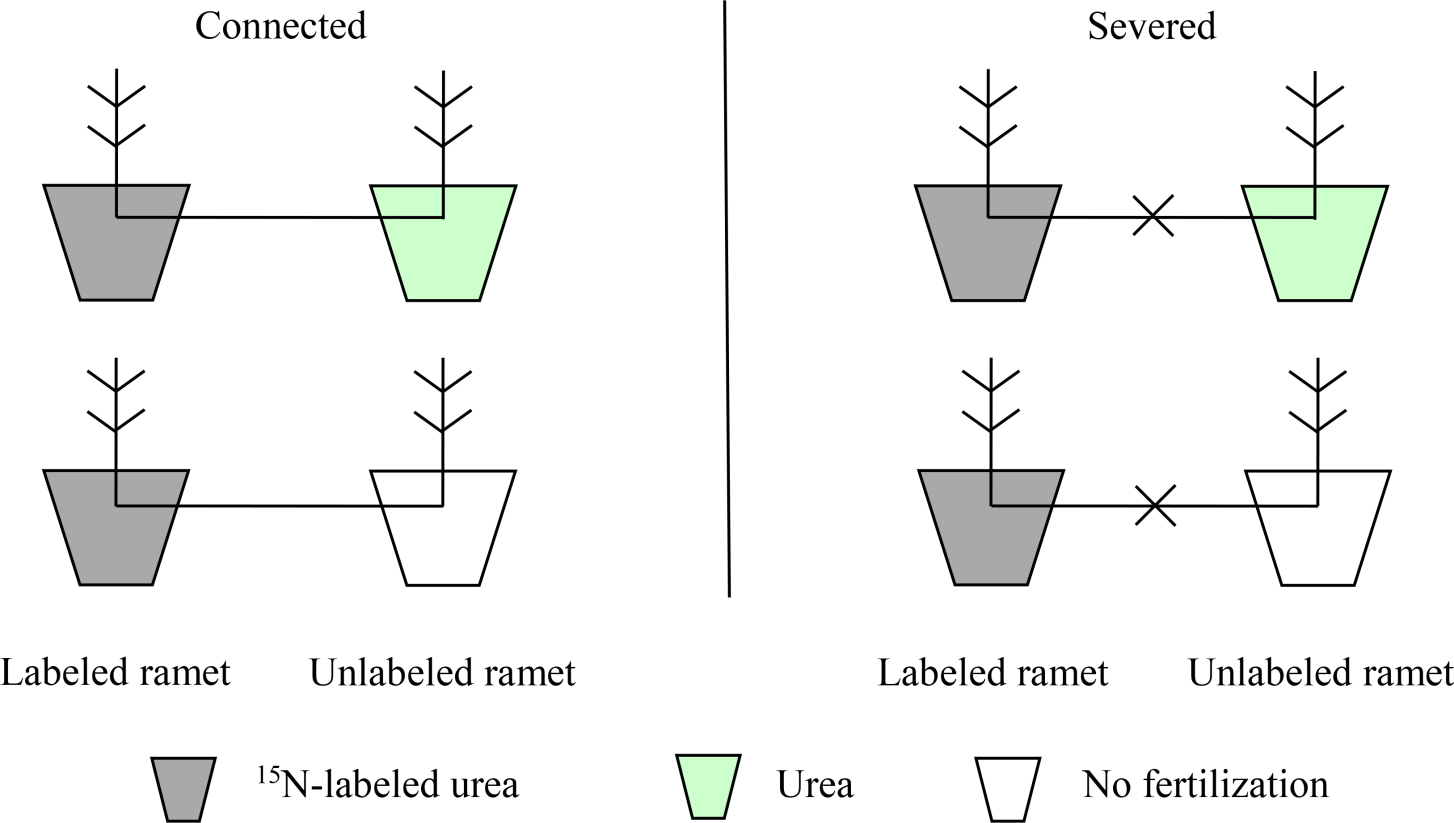
Schematic diagram of the experiment design.

In June 2022, 24 clonal fragments with relatively consistent growth were selected, which were divided into two groups (connected and severed). For the severed group, rhizomes between the two connected ramets were cut at the mid-point. In each group, two contrasting treatment (homogeneous and heterogeneous environments) were applied (**Figure 1**). In homogeneous N environment, one ramet of each clonal fragment was applied 20 g ^15^N-labeled urea, while the other ramet was applied 20 g urea. In heterogeneous N environment, one ramet of each clonal fragment was applied 20 g ^15^N-labeled urea, while the other ramet was not fertilized. Six replicates for each treatment were randomly arranged. The ^15^N-labeled urea (10.18 at%) was provided by Shanghai Research Institute of Chemical Industry, China. Fertilizers were irrigated into the soil as an aqueous solution in June 2021.

### 2.2 Samples collection and analysis

In October 2022, rhizomes were cut at the mid-point of the two connected ramets. Then, each ramet was harvested and separated into leaves, culms (including branches) and rhizomes (including rhizome roots). The soil particles attached to the rhizome and rhizome root were washed with running water. All the samples were dried at 65 °C in an oven (OF-12G, Lab Companion, South Korea) to a constant mass for biomass determination. Then, the dried samples were ground and sieved (100 mash). The total N concentrations and the at% ^15^N were determined using an Isotope Ratio Mass Spectrometer (Delta V Advantage, USA) at Institute of Soil Science, Chinese Academy of Sciences.

### 2.3 Calculation methods

Ndff (the percentage of ^15^N derived from ^15^N-labeled urea, %) was calculated by the following equation (Su et al., 2019):


$$\text{Ndff }\left(\%\right)=\frac{b-a}{c-a}\times 100$$

where a is the at% ^15^N of the unlabeled ramet in severed treatment at heterogeneous condition, b is the at% ^15^N of all the sampling ramets, and c is the at% ^15^N of ^15^N-labeled urea (10.18 at%).


$${\text{Organ}}^{15}\text{N concentration}\left({\text{g kg}}^{-1}\right)=\text{Organ N concentration}\left({\text{g kg}}^{-1}\right)\times \text{Ndff}\times 10^{-2}$$

$${\text{Organ}}^{15}\text{N}\left(\text{g}\right)=\text{Organ biomass}\left(\text{g}\right)\times {\text{Organ}}^{15}\text{N concentration}\left({\text{g kg}}^{-1}\right)\times 10^{-3}$$

$${\text{Ramet}}^{15}\text{N}\left(\text{g}\right)={\text{Leaf}}^{15}\text{N}\left(\text{g}\right)+{\text{Culm}}^{15}\text{N}\left(\text{g}\right)+{\text{Rhizome}}^{15}\text{N}\left(\text{g}\right)$$

$$\text{NUE}\left(\%\right)=\left({\text{Labeled ramet}}^{15}\text{N}+{\text{Unlabeled ramet}}^{15}\text{N}\right)\left(\text{g}\right)/{\text{Fertilizer}}^{15}\text{N}\left(\text{g}\right)\times 100$$

The ratio of the amount of ^15^N translocated from labeled ramet to unlabeled ramet to the amount of ^15^N absorbed by the clonal fragment was calculated to represent the intensity of physiological integration (IPI, %) according to the following equation (Saitoh et al., 2006):


$$\text{IPI }\left(\%\right)=\frac{AM_{i}}{AM_{frag}}\times 100$$

where AM_i_ is the amount of ^15^N translocated from labeled ramet to unlabeled ramet, and AM_frag_ is the total amount of ^15^N absorbed per clonal fragment.

The percentage increase of NUE in connected treatment compared with that in severed treatment was used to indicate the contribution rate of physiological integration (CPI, %).


$$\text{CPI }\left(\%\right)=\frac{NUE_{con}-NUE_{sev}}{NUE_{sev}}\times 100$$

where NUE_con_ is the NUE in connected treatment, and NUE_sev_ is the NUE in severed treatment.

### 2.4 Statistical analysis

Two-way analysis of variance (ANOVA) was used to investigate effects of rhizome connection status (connected vs. severed) and environment status (homogeneous vs. heterogeneous) on biomass, ^15^N concentrations and uptakes of ramets, and biomass, ^15^N uptakes and NUE of the whole clonal fragments. One-way analysis of variance (ANOVA) and Duncan’s multiple comparisons were used to analyze the significant differences between labeled and unlabeled ramets. In addition, differences in IPI and CPI between homogeneous and heterogeneous conditions were analyzed by One-way ANOVA. All analyses were conducted using SAS 9.4 software. Figures were prepared using Origin 8.6 software.

## 3 Results

### 3.1 Biomass accumulation of the ramets and the clonal fragment

**Figure 2:**
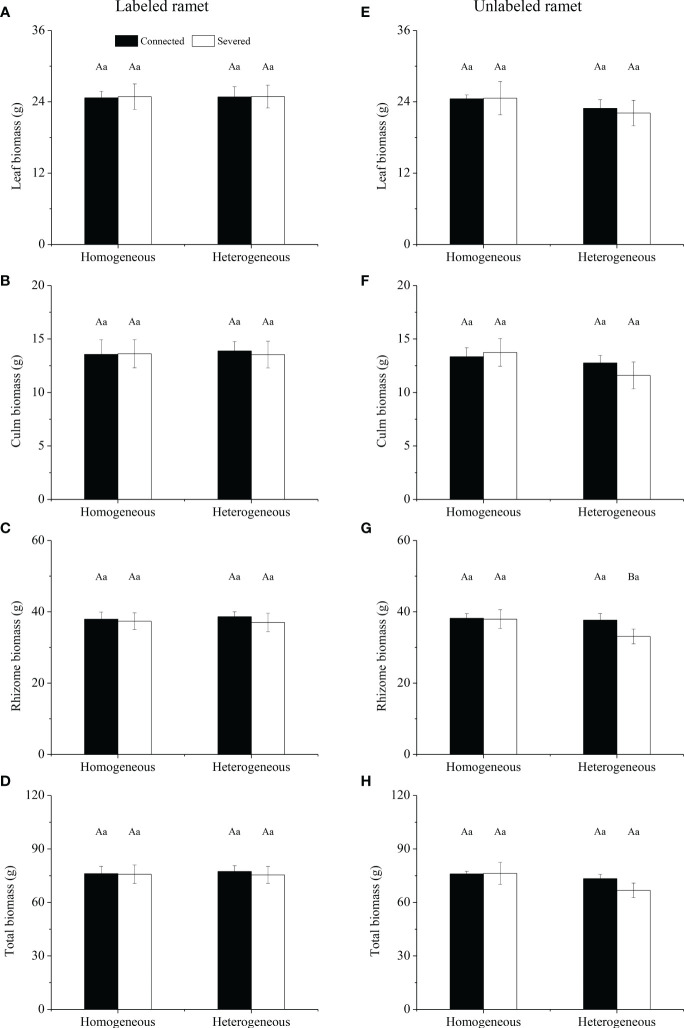
Leaf biomass **(A, E)**, culm biomass **(B, F)**, rhizome biomass **(C, G)** and total biomass **(D, H)** of the labeled **(A–D)** and the unlabeled **(E–H)** ramets in homogeneous and heterogeneous environments. The labeled and unlabeled ramets were either connected or severed. Different uppercase letters in each subgraph indicate statistically significant differences (*P<* 0.05) among the four treatments, and different lowercase letters indicate statistically significant differences (*P<* 0.05) between labeled and unlabeled ramets under the same treatment.

For labeled ramet, no significant effects of rhizome status, environment status and their interaction on leaf biomass, culm biomass, rhizome biomass and total biomass were observed (**Table 1**). No significant differences were found in leaf biomass, culm biomass, rhizome biomass and total biomass among the four treatment (**Figures 2A–D**). For unlabeled ramet, significant effect of environment status on total biomass was observed (**Table 1**). Leaf biomass, culm biomass and total biomass were not significantly different among the four treatments (**Figures 2E, F, H**). However, rhizome biomass of severed treatment under heterogeneous conditions was significantly lower than that of other treatments (*P*< 0.05, **Figure 2G**). Additionally, no significant differences were found in leaf biomass, culm biomass, rhizome biomass and total biomass between labeled and unlabeled ramets under the same treatment (**Figure 2**).

**Table 1:** Results of significance test for biomass, ^15^N concentrations and uptakes of labeled and unlabeled ramets.

	Organ	Labeled Ramet			Unlabeled Ramet		
		Rhizome status (R)	Environment status (E)	R×E	Rhizome status (R)	Environment status (E)	R×E
Biomass	Leaf	0.02	0.01	0.00	0.09	3.39	0.16
	Culm	0.04	0.03	0.08	0.39	5.06	1.65
	Rhizome	0.78	0.02	0.19	4.30	5.30	3.48
	Total	0.19	0.02	0.10	1.88	7.12*	0.17
^15^N concentration	Leaf	0.56	0.33	3.77	565.39***	220.84***	220.84***
	Culm	0.03	0.58	1.07	1071.73***	440.16***	440.16***
	Rhizome	0.22	0.01	0.02	970.86***	505.07***	505.07***
^15^N uptake	Leaf	0.23	0.05	0.85	207.09***	75.79***	75.79***
	Culm	0.01	0.24	0.66	713.74***	281.47***	281.47***
	Rhizome	0.00	0.01	0.06	845.62***	434.88***	434.88***
	Total	0.01	0.02	1.05	606.64***	264.32***	264.32***

Values are F ratio and significance are indicated by ***(P< 0.001), *(P< 0.05).

The effects of rhizome status (connected vs. severed), environment status (homogeneous vs. heterogeneous) and their interaction were tested in two-way ANOVA.

**Figure 3:**
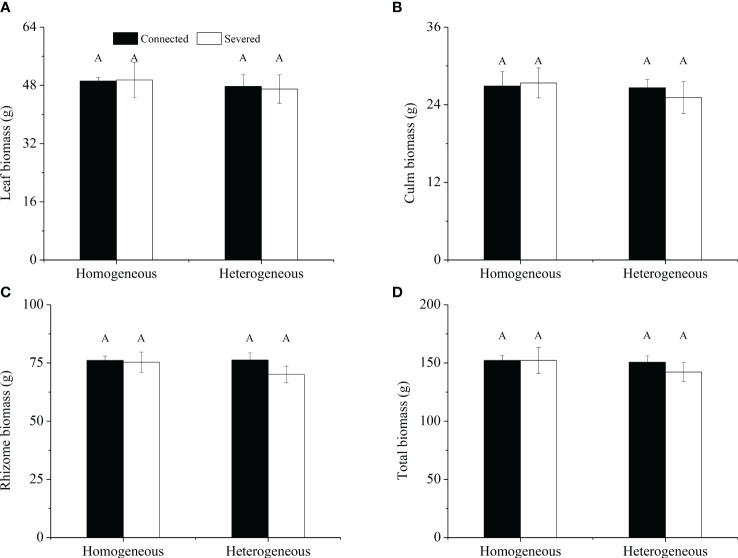
Leaf biomass **(A)**, culm biomass **(B)**, rhizome biomass **(C)** and total biomass **(D)** of the clonal fragment in homogeneous and heterogeneous environments. Different uppercase letters in each subgraph indicate statistically significant differences (*P<* 0.05) among the four treatments.

For the whole clonal fragment, no significant effects of rhizome status, environment status and their interaction on leaf biomass, culm biomass, rhizome biomass and total biomass were observed (**Table 2**). No significant differences were found in leaf biomass, culm biomass, rhizome biomass and total biomass among the four treatments (**Figure 3**). In heterogeneous conditions, leaf biomass, culm biomass, rhizome biomass and total biomass under the connected treatment were higher than those under the severed treatment, but no significant differences were observed (**Figure 3**).

**Table 2:** Results of significance test for biomass and ^15^N uptakes of the whole clonal fragments.

	Organ	Rhizome status (R)	Environment status (E)	R×E
Biomass	Leaf	0.01	0.93	0.06
	Culm	0.18	1.04	0.65
	Rhizome	3.27	1.69	1.97
	Total	0.90	1.66	0.90
^15^N uptake	Leaf	3.82	1.56	0.33
	Culm	7.35*	4.78	6.33*
	Rhizome	15.97**	8.82*	9.71*
	Total	8.77*	4.43	3.30

Values are F ratio and significance are indicated by **(P< 0.01), *(P< 0.05).

The effects of rhizome status (connected vs. severed), environment status (homogeneous vs. heterogeneous) and their interaction were tested in two-way ANOVA.

### 3.2 ^15^N concentrations of the ramets

**Figure 4:**
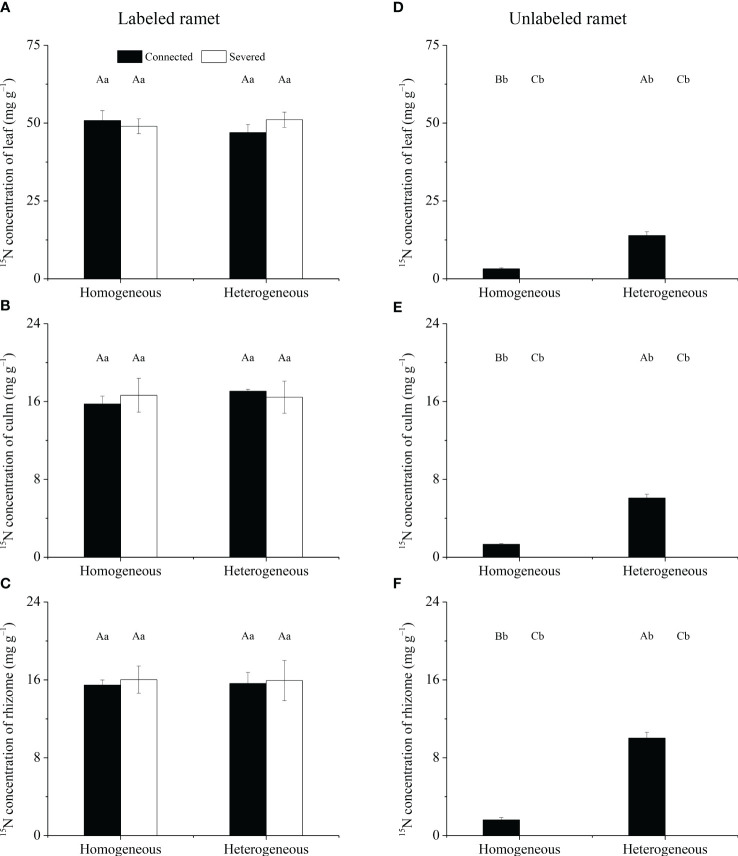
The ^15^N concentrations in leaf **(A, D)**, culm **(B, E)** and rhizome **(C, F)** of the labeled **(A–C)** and unlabeled **(D–F)** ramets in homogeneous and heterogeneous environments. The labeled and unlabeled ramets were either connected or severed. Different uppercase letters in each subgraph indicate statistically significant differences (*P<* 0.05) among the four treatments, and different lowercase letters indicate statistically significant differences (*P<* 0.05) between labeled and unlabeled ramets under the same treatment.

For labeled ramet, no significant effects of rhizome status, environment status and their interaction on ^15^N concentrations in leaf, culm and rhizome were observed (**Table 1**). ^15^N concentrations in leaf, culm and rhizome were not significantly different among the four treatments (**Figures 4A–C**). For unlabeled ramet, significant effects of rhizome status, environment status and their interaction on ^15^N concentrations in leaf, culm and rhizome were observed (**Table 1**). ^15^N concentrations in leaf, culm and rhizome of connected treatment in heterogeneous conditions were significantly higher than those in homogeneous conditions (*P*< 0.05), while these variables of severed treatment had no significant difference between homogeneous and heterogeneous conditions (**Figures 4D–F**). The ^15^N concentrations in leaf, culm and rhizome of connected treatment were significantly higher than those of severed treatment in both homogeneous conditions and heterogeneous conditions (*P*< 0.05, **Figures 4D–F**). In addition, the ^15^N concentrations in leaf, culm and rhizome of labeled ramet were significantly higher than those of unlabeled ramet under the same treatment (*P*< 0.05, **Figure 4**).

### 3.3 ^15^N uptakes of the ramets and the clonal fragment

**Figure 5:**
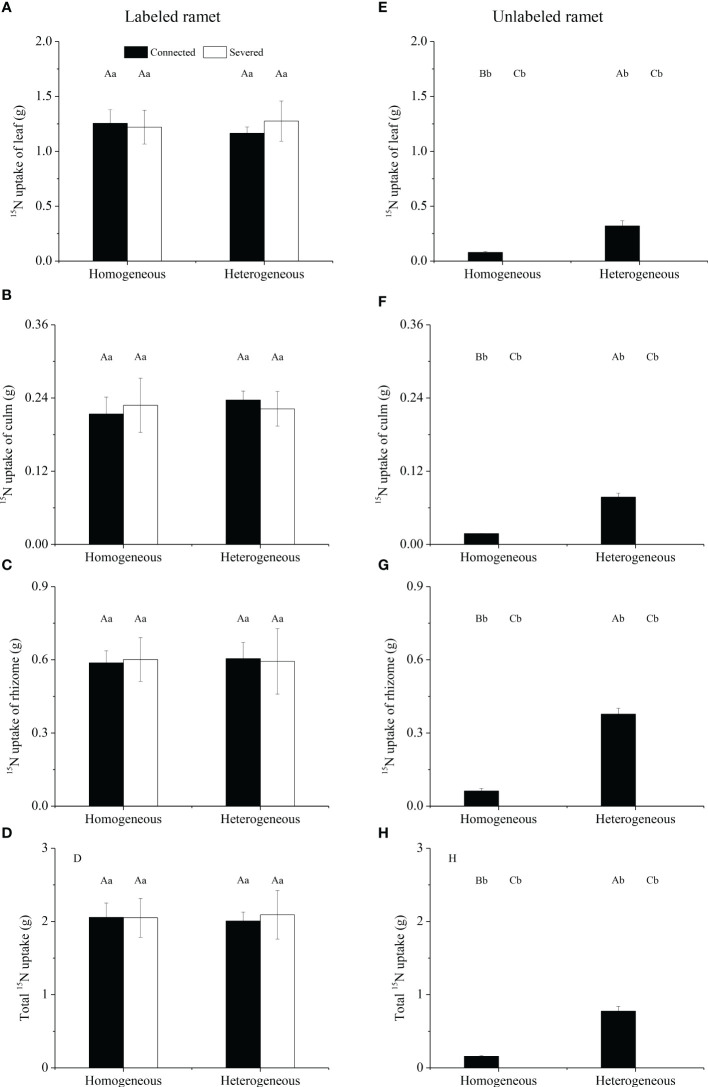
The ^15^N uptakes in leaf **(A, E)**, culm **(B, F)**, rhizome **(C, G)** and total **(D, H)** of the labeled **(A–D)** and unlabeled **(E–H)** ramets in homogeneous and heterogeneous environments. The labeled and unlabeled ramets were either connected or severed. Different uppercase letters in each subgraph indicate statistically significant differences (*P<* 0.05) among the four treatments, and different lowercase letters indicate statistically significant differences (*P<* 0.05) between labeled and unlabeled ramets under the same treatment.

For labeled ramet, no significant effects of rhizome status, environment status and their interaction on ^15^N uptakes in leaf, culm and rhizome were observed (**Table 1**). ^15^N uptakes in leaf, culm, rhizome and the total ^15^N uptake were not significantly different among the four treatments (**Figures 5A–D**). For unlabeled ramet, significant effects of rhizome status, environment status and their interaction on ^15^N concentrations in leaf, culm and rhizome were observed (**Table 1**). ^15^N uptakes in leaf, culm, rhizome and the total ^15^N uptake of connected treatment in heterogeneous conditions were significantly higher than those in homogeneous conditions (*P*< 0.05), while these variables of severed treatment had no significant difference between homogeneous and heterogeneous conditions (**Figures 5E–H**). The ^15^N uptakes in leaf, culm, rhizome and the total ^15^N uptake of connected treatment were significantly higher than those of severed treatment in both homogeneous and heterogeneous conditions (*P*< 0.05, **Figures 5E–H**). In addition, the ^15^N uptakes in leaf, culm, rhizome and the total ^15^N uptake of labeled ramet were significantly higher than those of unlabeled ramet under the same treatment (*P*< 0.05, **Figure 5**).

**Figure 6:**
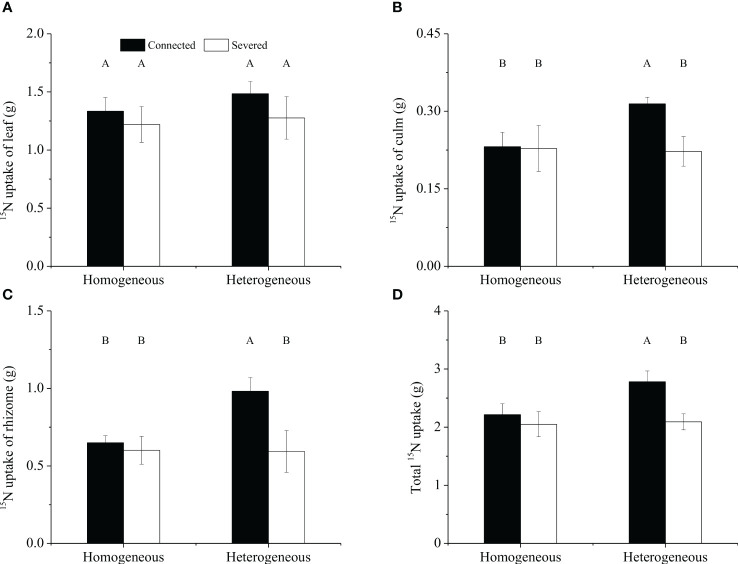
The ^15^N uptakes in leaf **(A)**, culm **(B)**, rhizome **(C)** and total **(D)** ^15^N uptake of the clonal fragment in homogeneous and heterogeneous environments. Different uppercase letters in each subgraph indicate statistically significant differences (*P<* 0.05) among the four treatments.

For the whole clonal fragment, no significant effect of rhizome status, environment status and their interaction on ^15^N uptakes in leaf was observed, whereas significant effect on ^15^N uptakes in rhizome was observed was found (**Table 2**). ^15^N uptakes in leaf was not significantly different among the four treatments (**Figure 6A**). However, the ^15^N uptakes in culm, rhizome and the total ^15^N uptake of connected treatment in heterogeneous environments were significantly higher than other treatments (*P*< 0.05, **Figures 6B–D**). What’s more, the total ^15^N uptake of connected fragment was higher than that of severed fragment in both homogeneous environments and heterogeneous environments (**Figure 6**).

### 3.4 Distributions of ^15^N in the connected clonal fragment

**Figure 7:**
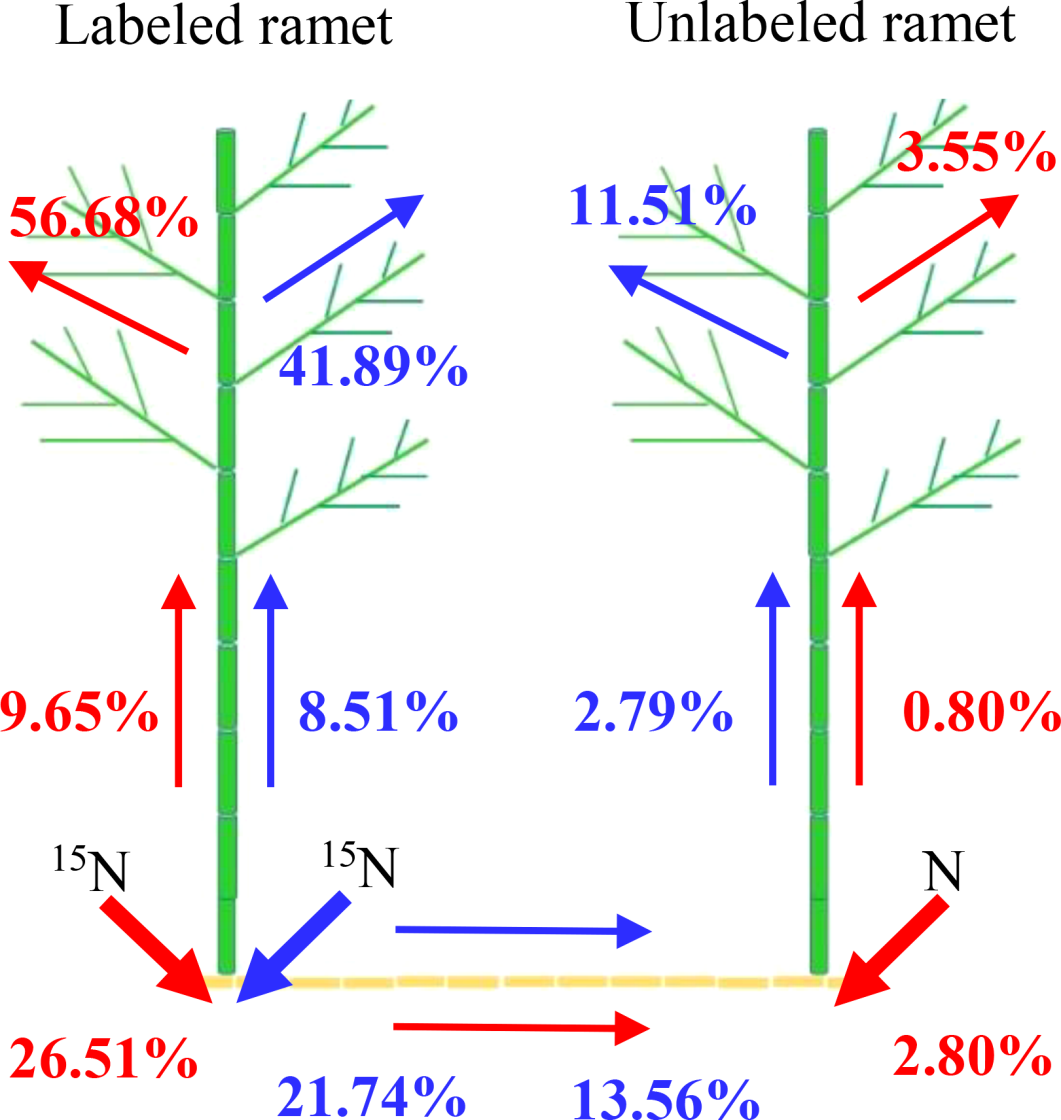
Distributions of the recovered ^15^N in the connected clonal fragment. Red arrows indicate ^15^N translocation and allocation in homogeneous environments, and blue arrows indicate ^15^N translocation and allocation in heterogeneous environments. The values (%) from top to bottom represent the proportion of ^15^N in leaf, culm and rhizome to the recovered ^15^N, respectively.

**Figure 8:**
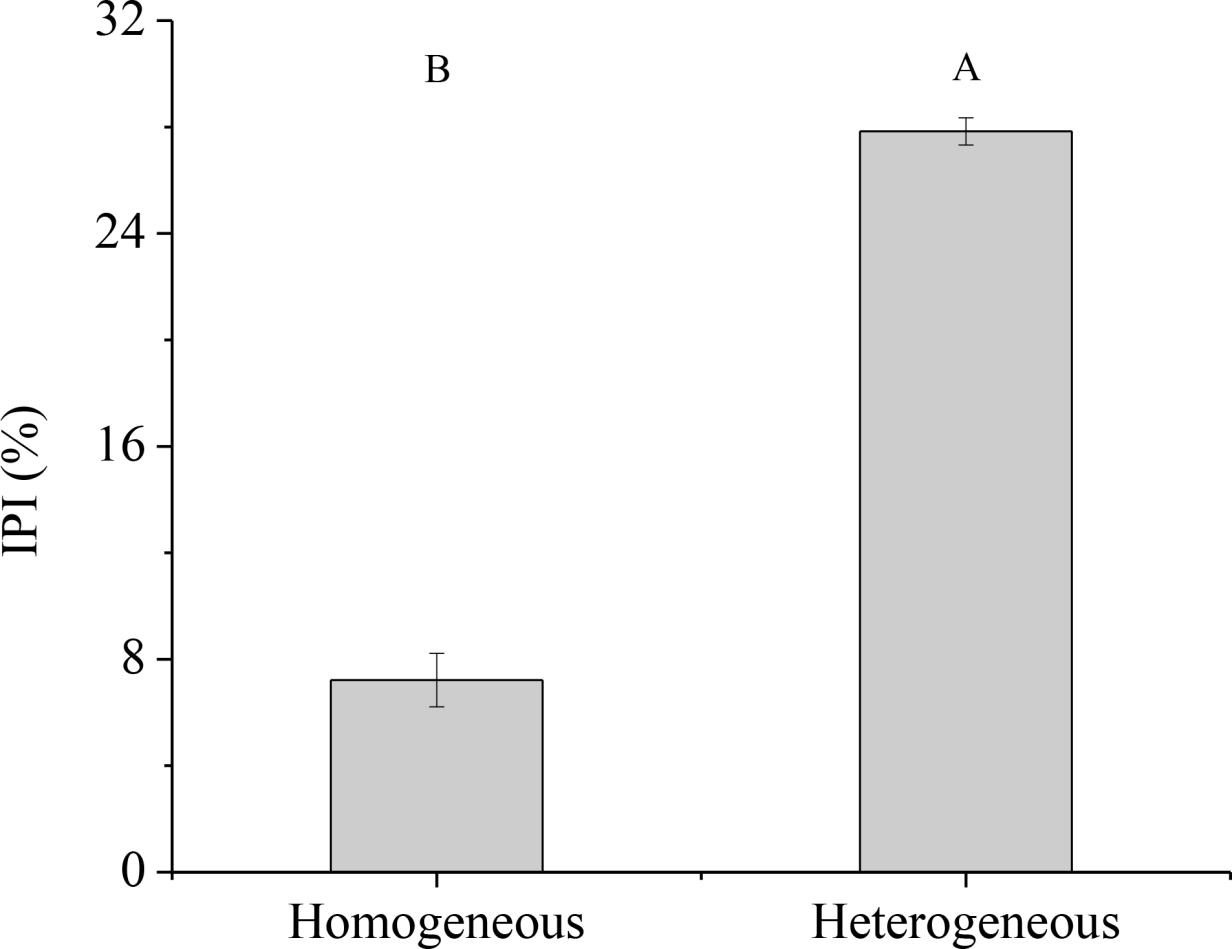
Intensity of physiological integration (IPI) of the connected clonal fragment in homogeneous and heterogeneous environments. Different uppercase letters indicate statistically significant differences (*P<* 0.05) between homogeneous and heterogeneous environments.

In homogeneous environments, the percentage of ^15^N absorption by unlabeled ramet was lower than the percentage of ^15^N absorption by labeled ramet (**Figure 7**). Approximately 92.85% of the recovered ^15^N was used by the labeled ramet, and its leaf, culm and rhizome retained 56.68%, 9.65% and 26.51% of ^15^N, respectively. In contrast, only 7.15% of the recovered ^15^N was moved to the unlabeled ramet, and its leaf, culm and rhizome retained 3.55%, 0.80% and 2.80% of ^15^N, respectively. In heterogeneous environments, when ^15^N-labeled urea was applied to one ramet, about 72.14% of the recovered ^15^N was stored in the labeled ramet, and the distributions of ^15^N in leaf, culm and rhizome were 41.89%, 8.51% and 21.74%, respectively. However, only 27.86% of the recovered ^15^N was moved to the connected ramet, and the distributions of ^15^N in leaf, culm and rhizome were 11.51%, 2.79% and 13.56%, respectively. As a result, the intensity of physiological integration (IPI) of the connected clonal fragments in heterogeneous environments was significantly higher than that in homogeneous environments (*P<* 0.05, **Figure 8**).

### 3.5 NUE of the clonal fragment

**Figure 9:**
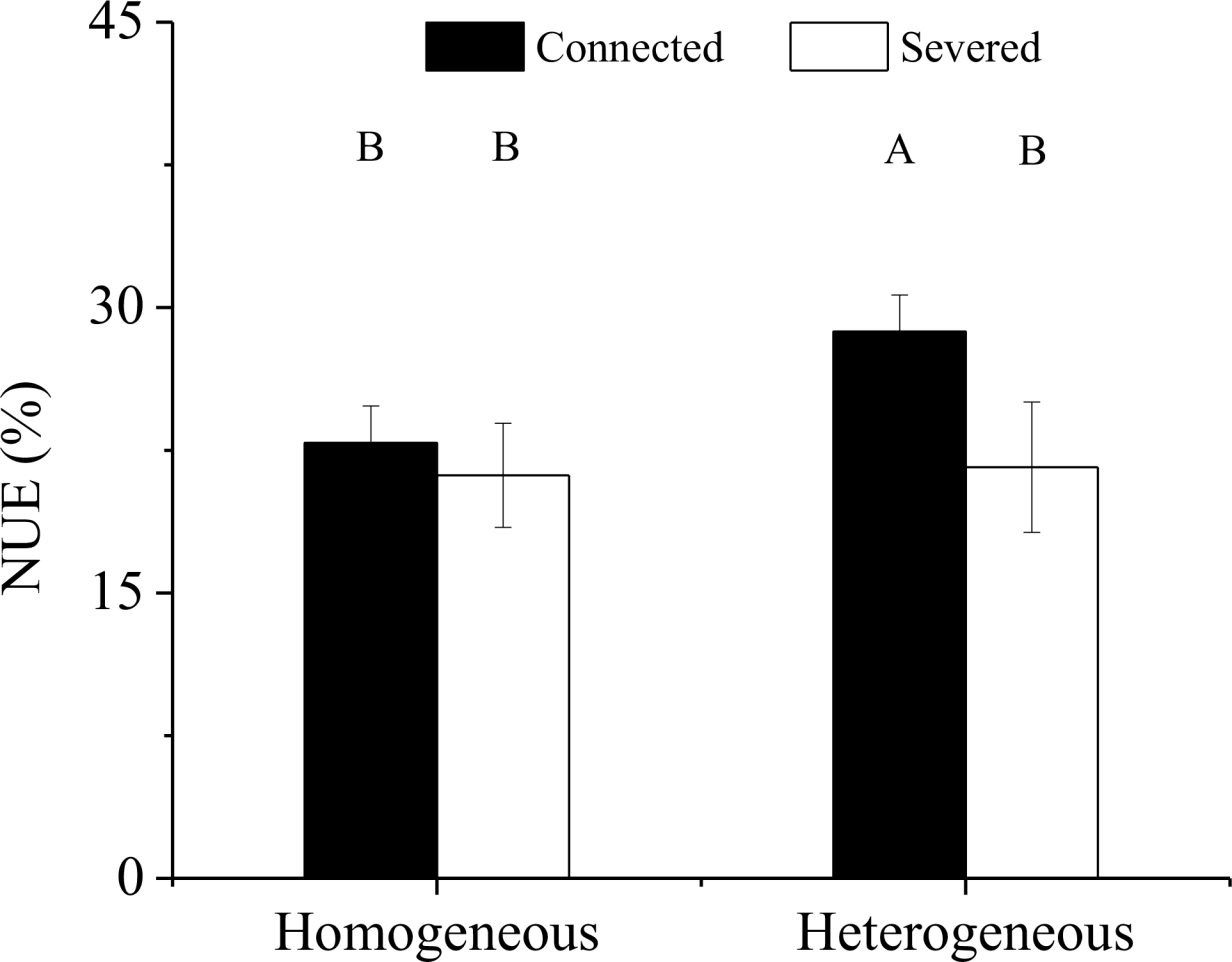
Nitrogen use efficiency (NUE) of the clonal fragment in homogeneous and heterogeneous environments. Different uppercase letters indicate statistically significant differences (*P<* 0.05) among the four treatments.

**Figure 10:**
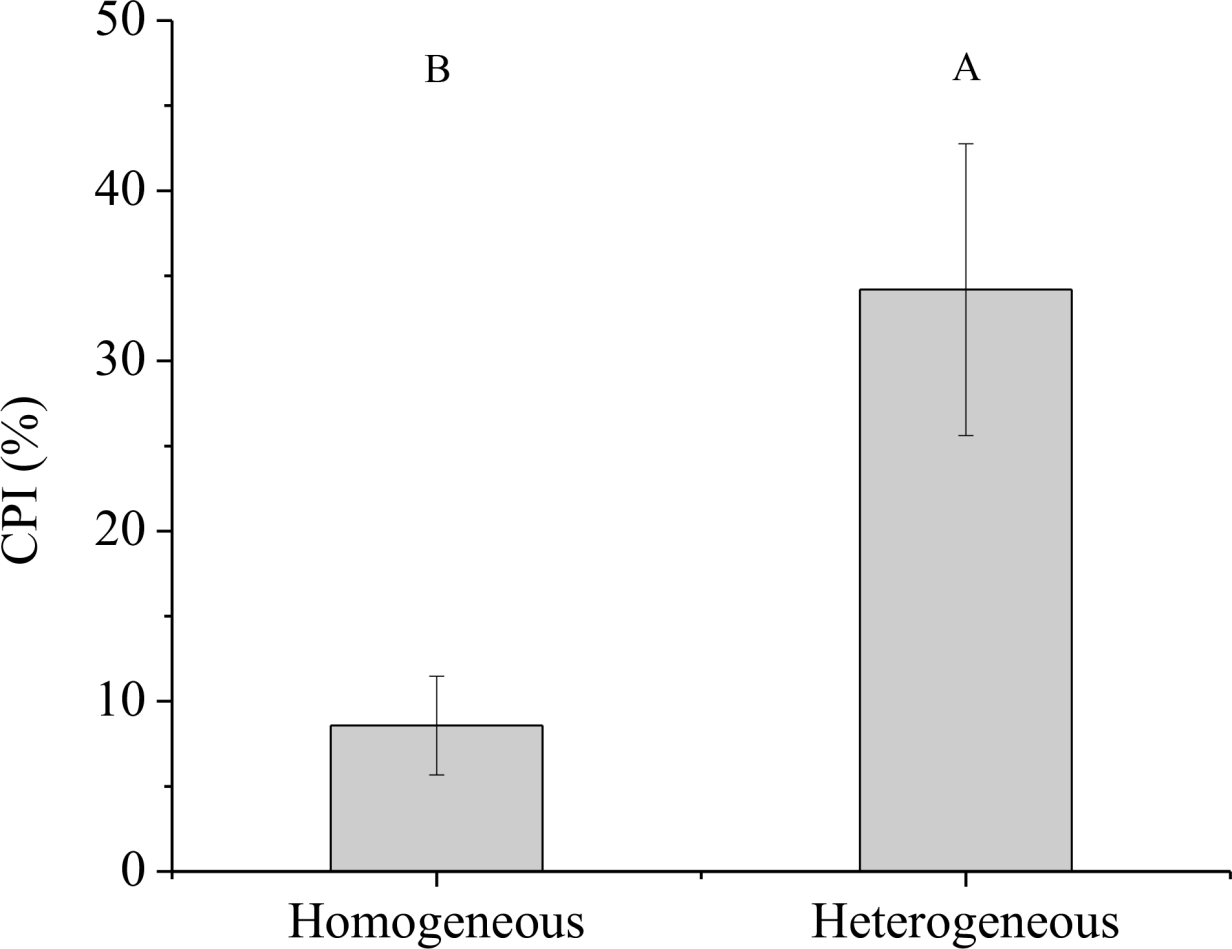
The contribution rate of physiological integration (CPI) of the connected treatments. Different uppercase letters indicate statistically significant differences (*P<* 0.05) between homogeneous and heterogeneous environments.

Significant effect of rhizome status on NUE was observed, while no significant effects of environment status and their interaction on NUE were found (**Table 3**). The NUE of the connected clonal fragment in heterogeneous environments was significantly higher than that in homogeneous environments (*P<* 0.05), while no significant difference was found in severed clonal fragment between homogeneous and heterogeneous environments (**Figure 9**). The NUE of the connected clonal fragment was significantly higher than that of the severed clonal fragment in heterogeneous environments (*P<* 0.05), while no significant difference was found in homogeneous environments (**Figure 9**). In addition, the contribution rate of physiological integration (CPI) in heterogeneous environments was significantly higher than that in homogeneous environments (*P<* 0.05, **Figure 10**).

**Table 3:** Results of significance test for NUE of the whole clonal fragments.

	Rhizome status (R)	Environment status (E)	R×E
NUE	8.77**	4.43	3.30

Values are F ratio and significance are indicated by **(P< 0.01).

The effects of rhizome status (connected vs. severed), environment status (homogeneous vs. heterogeneous) and their interaction were tested in two-way ANOVA.

## 4 Discussion

This study investigated the effects of physiological integration on N translocation and NUE of moso bamboo in homogenous and heterogeneous environments. However, the performance of N translocation and NUE was inconsistent in homogeneous and heterogeneous environments.

Previous studies showed that habitat heterogeneity was the external driving force for spatial expansion of clonal plant, and physiological integration was the internal driving force for clonal growth in heterogeneous environments (Liu, 2011). In addition, physiological integration was affected by the source-sink relationship between the connected clonal ramets, and the resource translocation was governed by sink strength (Chen et al., 2015; Dong et al., 2015; Shi et al., 2021; Shi et al., 2022). In this study, ^15^N translocation between labeled ramet and unlabeled ramet within clonal fragments of moso bamboo was found in heterogeneous environments (**Figure 7**). This discovery clearly revealed that moso bamboo was able to transfer ^15^N between labeled ramet and unlabeled ramet through rhizome in heterogeneous environments. When ^15^N-labeled urea was applied into one ramet (labeled ramet), approximately 27.86% of the absorbed ^15^N was transferred to the other ramet (unlabeled ramet), and 72.14% of the absorbed ^15^N was allocated to itself for growth. In other words, ramet grown under high-nutrient conditions could increase ^15^N uptakes as a compensatory response to meet the demand of the other ramet grown under low-nutrient conditions (He et al., 2010). This result indicated that the ^15^N allocation of fertilized ramet (labeled ramet) was higher than the connected unfertilized ramet (unlabeled ramet) for the growth of itself. The phenomenon with low rates of resource sharing was also found in *Fragaria chiloensis* in heterogeneous environments, which was called “selfish” (Alpert, 1999). Therefore, the fertilized ramet could transmit nutrients to the connected unfertilized ramet through physiological integration after meeting its own growth, and then improve the adaptability of the clonal fragment in heterogeneous environments.

Numerous studies showed that clonal plants rarely carried out physiological integration or refused physiological integration in homogenous environments (de Kroon et al., 1996; Alpert, 1999; Gao et al., 2013). In the present study, ^15^N translocation between labeled ramet and unlabeled ramet within clonal fragments of moso bamboo was also found in homogenous environments (**Figure 7**). This discovery clearly revealed that moso bamboo was able to transfer ^15^N between labeled ramet and unlabeled ramet through underground rhizome-root system in homogenous environments. When ^15^N-labeled urea was applied into one ramet (labeled ramet), only 7.15% of the absorbed ^15^N was transferred to the other ramet (unlabeled ramet), and 92.85% of the absorbed ^15^N was allocated to itself for growth. This phenomenon suggested that N transfer occurred between the connected ramets within clonal fragment of moso bamboo in homogeneous environments, but the amount was relatively low. In other words, the unlabeled ramets did not require a lot of N supply from labeled ramets in homogeneous environments, which could decrease the cost of survival and maintain their internal balance (Gao et al., 2013).In both homogenous and heterogeneous environments, physiological integration significantly increased the ^15^N uptakes of the unlabeled ramets (**Figure 5**), which was potentially able to increase the total ^15^N uptakes and then improve the NUE of the clonal fragments. Physiological integration had no significant effect on the total ^15^N uptakes within the clonal fragments between connected and severed treatment in homogenous environments, while the total ^15^N uptake of connected treatment was significantly higher than that of severed treatment in heterogeneous environments (**Figure 6**). Although the source-sink relationship was the driving force for physiological integration (Shi et al., 2021), physiological integration also occurred in homogeneous environments, with a lower intensity of physiological integration compared to heterogeneous environments (**Figure 8**).

Physiological integration has been considered an important factor in relation to altering nutrient use efficiency in clonal plants (He et al., 2010; Shi et al., 2022). In this study, we found that the NUE of the connected clonal fragment was significantly higher than that of the severed fragment in both homogenous and heterogeneous environments (**Figure 9**). This result indicated that there was a close link between NUE and physiological integration. In heterogeneous environments, ramets grown in low-nutrient conditions could obtain ^15^N from high-nutrient conditions through physiological integration, thus improving the NUE of the whole clonal fragment. Although the ^15^N uptakes of labeled ramets decreased, the ^15^N uptakes of unlabeled ramets increased in both homogenous and heterogeneous environments, and the increase in the unlabeled ramet was much greater than the reduction in the labeled ramet (**Figure 5**). As a result, a net benefit of physiological integration to the whole clonal fragment was found (Wang et al., 2021), which improved the NUE of moso bamboo. However, the contribution rate of physiological integration (CPI) on NUE was not consistent between homogenous and heterogeneous environments, and the difference was significant. Our results also indicated that physiological integration was more likely to occur in heterogeneous environments, while it rarely occurred in homogeneous environments.

## 5 Conclusion

This study clearly revealed the relationship between physiological integration and N translocation and NUE of moso bamboo in homogenous and heterogeneous environments. The N translocation between the connected ramtes of moso bamboo was determined by the source-sink relationship in heterogeneous environments. Physiological integration was also occurred in homogeneous environments, but its intensity was relatively low. There was a close relationship between physiological integration and NUE, and physiological integration significantly improved the NUE of moso bamboo in both homogeneous and heterogeneous environments. These results will provide theoretical basis for precision fertilization in moso bamboo forests.

## Data availability statement

The original contributions presented in the study are included in the article/supplementary material. Further inquiries can be directed to the corresponding authors.

## Author contributions

JZ: Conceptualization, Investigation, Formal analysis, Writing-original draft, Funding acquisition. CC: Conceptualization, Formal analysis, Writing-review & editing, Supervision, Funding acquisition. All authors contributed to the article and approved the submitted version.
